# SWIP—a stabilized window for intravital imaging of the murine pancreas

**DOI:** 10.1098/rsob.210273

**Published:** 2022-06-15

**Authors:** Wei Du, Christian Adkisson, Xianjun Ye, Camille L. Duran, Benson Chellakkan Selvanesan, Claudia Gravekamp, Maja H. Oktay, John C. McAuliffe, John S. Condeelis, Nicole C. Panarelli, Robert J. Norgard, Yogev Sela, Ben Z. Stanger, David Entenberg

**Affiliations:** ^1^ Breast Center, Peking University People's Hospital, Beijing, People's Republic of China; ^2^ Anatomy and Structural Biology, Einstein College of Medicine/Montefiore Medical Center, Bronx, NY, USA; ^3^ Department of Cell Biology, Einstein College of Medicine/Montefiore Medical Center, Bronx, NY, USA; ^4^ Department of Surgery, Einstein College of Medicine/Montefiore Medical Center, Bronx, NY, USA; ^5^ Gruss-Lipper Biophotonics Center, Einstein College of Medicine/Montefiore Medical Center, Bronx, NY, USA; ^6^ Integrated Imaging Program, Einstein College of Medicine/Montefiore Medical Center, Bronx, NY, USA; ^7^ Department of Pathology, Einstein College of Medicine/Montefiore Medical Center, Bronx, NY, USA; ^8^ Department of Microbiology and Immunology, Einstein College of Medicine/Montefiore Medical Center, Bronx, NY, USA; ^9^ Department of Medicine, Perelman School of Medicine, University of Pennsylvania, Philadelphia, PA, USA

**Keywords:** intravital imaging, pancreatic ductal adenocarcinoma, pancreatitis, cerulein

## Abstract

Pancreatitis and pancreatic ductal adenocarcinoma (PDAC) are grave illnesses with high levels of morbidity and mortality. Intravital imaging (IVI) is a powerful technique for visualizing physiological processes in both health and disease. However, the application of IVI to the murine pancreas presents significant challenges, as it is a deep, compliant, visceral organ that is difficult to access, easily damaged and susceptible to motion artefacts. Existing imaging windows for stabilizing the pancreas during IVI have unfortunately shown poor stability for time-lapsed imaging on the minutes to hours scale, or are unable to accommodate both the healthy and tumour-bearing pancreata. To address these issues, we developed an improved stabilized window for intravital imaging of the pancreas (SWIP), which can be applied to not only the healthy pancreas but also to solid tumours like PDAC. Here, we validate the SWIP and use it to visualize a variety of processes for the first time, including (1) single-cell dynamics within the healthy pancreas, (2) transformation from healthy pancreas to acute pancreatitis induced by cerulein, and (3) the physiology of PDAC in both autochthonous and orthotopically injected models. SWIP can not only improve the imaging stability but also expand the application of IVI in both benign and malignant pancreas diseases.

## Graphical abstract

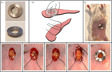

## 1. Introduction

Benign and malignant pancreatic diseases such as pancreatitis and pancreatic ductal adenocarcinoma (PDAC) are serious life-threatening disorders. Pancreatitis is the leading cause for gastrointestinal (GI) disease-related hospital admissions [1] and PDAC, accounting for over 90% of pancreatic malignancies [2], is the fourth leading cause of cancer death in the USA, with a poor 5-year survival rate of only approximately 9% [3]. Since PDAC is mainly asymptomatic during its initial stages, it often presents at a late stage, resulting in limited treatment options for most patients. Therefore, understanding pancreatic diseases is crucial for improving patient prognosis.

Intravital imaging (IVI) enables the visualization and analysis of tumour cell dynamics in live animals and in real time. Previous research has used IVI to observe benign and malignant diseases of skin [4,5], breast [6], lung [7], liver [8], brain [9] and pancreatic tumours [10]. However, the murine pancreas presents significant challenges to single-cell resolution IVI as it is a deep visceral organ and is extremely compliant. Furthermore, it is a diffusely distributed branched organ within the mesentery that attaches to the stomach, spleen and small intestine. These features make the tissue both difficult to access, and easily affected by both the nearby peristalsis and the constant movement of respiration [11,12]. Minimizing this movement is essential for high-resolution microscopy, as motion artefacts of even a few microns can blur and distort images or make tracking of individual cells over time impossible [13].

To overcome these limitations, previous studies have used different implantable imaging window designs. Ritsma *et al.* [8] were the first to succeed in establishing a window system to access internal organs for IVI. Called the abdominal imaging window (AIW), this system created a portal to the visceral organs using a titanium window frame sutured into the abdominal wall. Tissue stabilization was accomplished by adhering the organ of interest to the titanium window frame with cyanoacrylate. The AIW was used to image snapshots of the pancreatic tumours every 15 min, employing software-based corrections to eliminate XY and Z drift [10]. While this worked well for some rigid internal organs (e.g. liver, spleen, pancreata bearing rigid tumours), suboptimal lateral and axial stability was appreciated in the more complaint, normal murine pancreas [14].

To address this issue, Park *et al.* [14] developed a new pancreas imaging window (PIW) designed specifically for pancreas imaging. By incorporating within the window frame a horizontal metal shelf that the pancreas can be rested upon, the influence of intestinal movement and breathing can be minimized. However, the PIW was limited by a suboptimal axial drift and imaging large solid tumours due to the narrow gap between the metal plate and the cover glass.

During our recent experience developing an implantable imaging window for thyroid tumours [15], we found that deep-set tissues can be stabilized by using a cross stitch ‘basket’ run underneath the tissue, effectively mimicking the ligaments used by the body to secure and stabilize organs. The use of this soft basket (which cradles, but does not penetrate, the tissue) both prevents lateral motion as well as supplies constant axial pressure, keeping the tissue against the glass. We previously used a ‘cross stitch’ technique to stabilize the murine thyroid for intravital imaging [15]. We sought to incorporate this technique here to overcome the limitations of the AIW and PIW in imaging the murine normal and diseased pancreas. This window, which we call the stabilized window for intravital imaging of the pancreas (SWIP), can be used for continuous imaging (up to 12 h, due to protocol limits) as well as for serial imaging over multiple consecutive days to track regions of interest over time. To show the utility of the SWIP, we use it to visualize a variety of processes for the first time, including (1) single-cell dynamics within the healthy pancreas, (2) transformation from healthy pancreas to acute pancreatitis induced by cerulein and (3) physiology of PDAC in both autochthonous and orthotopically injected models.

## 2. Material and methods

### 2.1. Animal models

Six variants of transgenic or autochthonous mice were used for intravital imaging: (1) MMTV-iCre/CAG-CAC-ECFP (ECFP labelled epithelia) used for imaging the mammary gland or the healthy pancreas; (2) FVB: MMTV-iCre/CAG-CAC-ECFP × c-fms-GFP [16] (ECFP labelled epithelia and GFP labelled macrophages) used for imaging of healthy pancreas; (3) C57B6: prox1-tdTomato × Csf1r-GAL4-VP16/UAS-ECFP [17] (tdTomato labelled lymphatic endothelia and ECFP labelled macrophages) used for orthotopic injections; (4) mixed: Kras^G12D^/p53^fl/+^/Pdx1-Cre/Rosa^YFP^ (KPCY, YFP labelled pancreata that spontaneously forms pancreatic tumours) [18]; (5) mixed: Pdx1-Cre/p53^fl/+^/Rosa^YFP^ (PCY, YFP labelled pancreata); and (6) mixed: Pdx1-Cre/Rosa^YFP^ (CY, YFP labelled pancreata). Mice were housed in static microisolator cages under specific pathogen-free conditions in a temperature- and humidity-controlled environment. All mice were provided with 1/8 inch corn cob as bedding material and Nestlets as nesting material. All experiments were performed during the daytime of a 12-hour day/night cycle. KPCY, PCY and CY mice were used between 16 and 20 weeks of age, when they develop palpable tumours. All others were born and raised within in the same animal housing facility and used between 8 and 12 weeks of age.

### 2.2. Transfection of KPC cells with Dendra2

KPC cells [19] were cultured (5% CO2 at 37°C) in McCoy's 5A medium (Gibco, 16600-082) with 10% FBS, 1% MEM non-essential amino acids (Gibco, 11140-050), 1% sodium pyruvate (Gibco, 11360-070) and 1% penicillin and streptomycin. Cells were seeded at 70% confluency in Opti-MEM I Reduced Serum Medium (Gibco, 31985-062) without added serum, in a six-well plate. Transient transfection was with 1.5 µg Dendra2-CMV plasmid and 12.5 µl Lipofectamine 2000 (ThermoFisher, 12566014). To create the stable KPC/Dendra2 cell line, after 24 h of transfection, the media was changed to complete media, and 24 more hours later, G418 (Gibco, 10131-035) was added 600 µg ml^−1^. Selection occurred for two weeks with 600 µg ml^−1^ G418 in.complete media, changing media every 2 days. After reaching confluency, the top 10–15% Dendra2 green expressing cells were collected using flow assisted cell sorting (FACS).

### 2.3. Cerulein treatment

The mice were fasted for 14–18 h with water given *ad libitum*. 50 µg kg^−1^ of cerulein in 100 µl sterile 1× DPBS was injected intraperitoneally at one-hour intervals for up to eight injections. Control mice received an equal volume of 1× DPBS. Mice were sacrificed 24 h after first injection. Pancreata were collected and fixed for 72 h in 10% formalin and paraffinized.

### 2.4. Immunofluorescence staining for cell apoptosis

Deparaffinized sections were blocked in 5% donkey serum for 1 h at room temperature, incubated with primary antibodies for overnight at 4°C. Sections were washed with PBST and incubated with secondary antibodies for 1 h at room temperature. Primary antibodies were chicken anti-GFP (Abcam, ab13970) at 1:250 and rabbit anti-cleaved caspase 3 (Cell Signaling Technology, CST9664). Lastly, sections were washed with PBST and mounted with Aquamount and DAPI. Slides were imaged on 3DHistech's Pannoramic 250 Flash II slide scanner.

### 2.5. Orthotopic injection

KPC-dendra2 cells were cultured in McCoy's 5A medium (Gibco, 16600-082) supplemented with 10% fetal bovine serum, 1% MEM non-essential amino acids (Gibco, 11140-050), 1% sodium pyruvate (Gibco, 11360-070) and 1% penicillin and streptomycin. Single-cell suspensions were prepared at a concentration of 1×10^6^ cells/50 µl PBS and kept on ice.

The orthotopic injection protocol was adapted from Erstad *et al*. [20]. Anaesthesia was induced using 5% isoflurane (Covetrus, 029405) and oxygen. Ophthalmic ointment (Dechra Veterinary Products, 17033-211-38) prevented corneal drying and abdomen was depilated. Mice were transferred to the sterile surgical field and placed in a right lateral decubitus position. Anaesthesia was lowered to 2% and the surgical site was sanitized with chlorhexidine (Durvet, Inc., 30798-624-31).

A 10–15 mm longitudinal incision was made in the left flank of the abdomen of the mouse, just below the left costal margin. The spleen, along with the attached tail of the pancreas, was brought out of the incision using cotton swabs. 50 µl of KPC-dendra2 cell suspension was slowly injected using a 28-gauge needle (Becton, Dickinson and Company, 329424) while holding the edge of the pancreas with atraumatic forceps. Successful injection was confirmed by the presence of a well-defined bubble within the pancreas. To prevent leakage, the needle was inserted parallel to the pancreas and slowly withdrawn over 30 s. The spleen and pancreas were returned to their anatomical position by gently retracting the skin and muscle flaps. Skin and musculature were closed using interrupted 5-0 silk suture. Surgical adhesive (3 M Company, 1469SB) was applied over the incision and sutures. Mice were observed throughout recovery. SWIP implantation occurred when tumours were approximately 0.5–1 cm.

### 2.6. Window passivation

**Figure 1.**
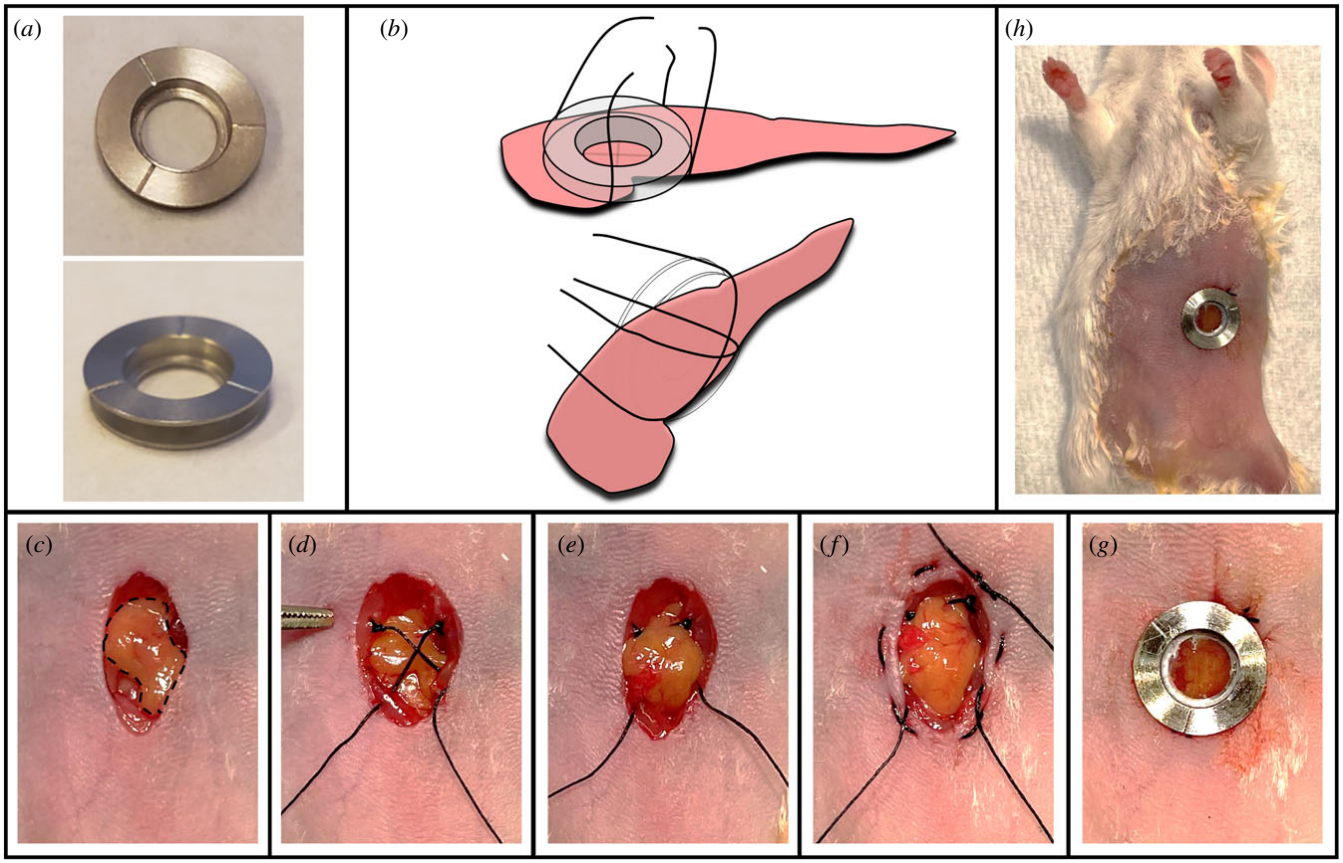
Overview of the stabilized window for imaging of the pancreas (SWIP) and SWIP surgical protocol. (*a*) Picture of the SWIP. Three lines etched on the frame allow the use of microcartography to relocate regions of interest during serial imaging. (*b*) Cartoon demonstrating the cross stitch ‘basket’ that stabilizes the pancreas tissue laterally and axially. (*c*–*g*) Summary of the steps of the SWIP surgical procedure. (*c*) An incision is made in the upper left quadrant of abdomen through the skin and abdominal wall and the pancreas exposed (dashed outline). (*d*) Cross stitch is placed over the pancreas and one end is secured to the abdominal muscle with a square knot. (*e*) The pancreas is gently laid on top of the cross stitch. (*f*) A purse-string suture is placed through the skin and abdominal wall around the incision. (*g*) The window frame is fitted in the incision with both the skin and abdominal wall within the frame's groove. The purse string suture is tightened and the free end of cross stitch pulled gently taut and tied in the groove. Finally, adhesive is applied to the recess of the window and cover glass affixed in place. (*h*) View of the mouse post window implantation.

The custom-made reusable stainless steel SWIP window (figure 1*a*) was passivated before each use, as previously published [7].

### 2.7. Intravital imaging

All imaging was performed on a previously described, custom-built, inverted two-laser multiphoton microscope [21]. All images were captured in 16 bit using a 25 × 1.05 NA objective lens (Olympus) using a tunable femtosecond laser (Spectra Physics, Mai-Tai) tuned to 880 nm, typically with two frame averages. Fluorescence and second-harmonic signals were separated via dichroic mirrors and collected using separate photomultiplier tubes, as previously described [21].

Mice were anaesthetized and ophthalmic ointment applied. Mice were injected retro-orbitally with 100 µl of 20 mg ml^−1^ 155kD tetramethyl-rhodamine dextran. A thin coating of petroleum jelly was applied to the underside of the microscope stage plate to prevent wicking of water from the objective lens [7]. A previously published window fixturing plate was used to immobilize the SWIP and secure the mouse to the imaging stage [7]. The fixturing plate was also coated with a thin layer of petroleum jelly and secured in place with paper tape. Mice were catheterized as described by Harney *et al.* [22]. 50 µl h^−1^ PBS was administered to prevent dehydration. Physiological temperature was maintained using a stage top environmental chamber. Vasculature of the tissue was identified by observing flowing erythrocytes and the tissue of interest identified by fluorescence. Vitals were monitored during imaging (Mouse Stat for PhysioSuite, Kent Scientific). A minimal amount of image processing was performed to optimize brightness and contrast of images.

### 2.8. Image analysis

Quantification of image drift and instability was performed using our previously published ROI_Tracker plugin for ImageJ [23] to mark identifiable structures within time lapse moves and quantify their location and displacement over time. All images were analysed in ImageJ/Fiji (NIH) [23].

## 3. Results

Given the compliant and delicate nature of the murine pancreas, as well as its relatively deep positioning compared to other abdominal organs, the pancreas can be challenging to immobilize for intravital imaging.

In using the AIW, we found that, even with adhesive applied to the inner side of the frame, a large amount of axial and lateral drift could be observed (electronic supplementary material, figure S1A and movie S1), and, as was reported by Park *et al*. [14], the tissue would disconnect from the frame and move within the window's clear aperture over time. The solution proposed by Park *et al*. (modifying the AIW to include a metal shelf within the window's aperture) braces the tissue and presses the pancreas against the cover glass so as to fill the entire clear aperture of the window, greatly increasing the volume of tissue available for observation. Upon testing this new window design, we indeed observed a markedly improved lateral stability. However, we still observed substantial axial drift over several hours as a result of tissue settling within the gap between the cover glass and the plate (electronic supplementary material, figure 1B and movie S2). Furthermore, although the integrated plate offered improved stability, its presence limited the ability to image solid pancreatic tumours, as their large size would not fit within the limited space between the plate and cover glass.

### 3.1. Development of the surgical protocol

In order to create a permanent, implantable optical-imaging window (figure 1*a*) that would allow stable time-lapsed imaging of both healthy and diseased pancreatic tissue, we developed the surgical protocol summarized in figure 1. To minimize the empty space seen in the AIW and the PIW and provide a larger stabilizing area around the imaged tissue, we chose a window frame with a 5 mm clear aperture (figure 1*a*). This window design, which we used previously for lung imaging [7], includes three etched lines on the frame which act as fiducial markers, allowing the use of microcartography [24] for relocalization of areas of interest during serial imaging [7]. While this improved stability during short term imaging, we still found the tissue susceptible to separating from the cover glass, especially while the animal was ambulatory. To address this, we turned to our previously published [15] cross stitch ‘basket’ technique (figure 1*b*) which mimics the body's use of ligaments to secure and stabilize organs. This soft basket (which cradles, but does not penetrate, the tissue) keeps the tissue apposed to the glass with constant axial pressure, preventing lateral and axial motion. For larger solid tumours, the support point and the direction of the cross stitch can be adjusted according to the size and position of the tumour for optimal support.

The surgery consists of a approximately 10 mm longitudinal incision made in the left upper quadrant of the abdomen. The skin and abdominal musculature are spread to expose the pancreas and spleen within a circular incision approximately 10 mm in diameter (figure 1*c*). The cross stitches (figure 1*b*) are placed with 5-0 silk suture where one end of each cross stitch is tied to the abdominal muscle and the free end left to form the ‘basket’ (figure 1*d*). The tail and body of the pancreas is then laid on top of the basket using sterile cotton swabs to avoid damaging the delicate tissue (figure 1*e*). A purse-string suture placed through the skin and abdominal wall secures the window frame to the abdomen (figure 1*f*). After placement of the SWIP frame on top of the pancreas, the skin and abdominal wall are placed in the groove of the window frame and the purse-string tightened. By ensuring that stitch steps are not greater than 5 mm, excessive skin folding can be avoided when tightening the purse string. The free ends of cross stitch are next tightened and tied in the groove. The open aperture of the window frame is gently dried with compressed air and a cover glass is affixed to the bottom of the bored recess of the frame using cyanoacrylate glue (figure 1*g*). This procedure reseals the abdominal cavity, prevents infection and effectively makes the window and the pancreas one monolithic unit (figure 1*h*). Using custom-made fixturing and stage plates [7] further stabilizes the imaged tissue relative to the objective lens.

### 3.2. The SWIP improves stability of imaging over both the AIW and PIW

**Figure 2.**
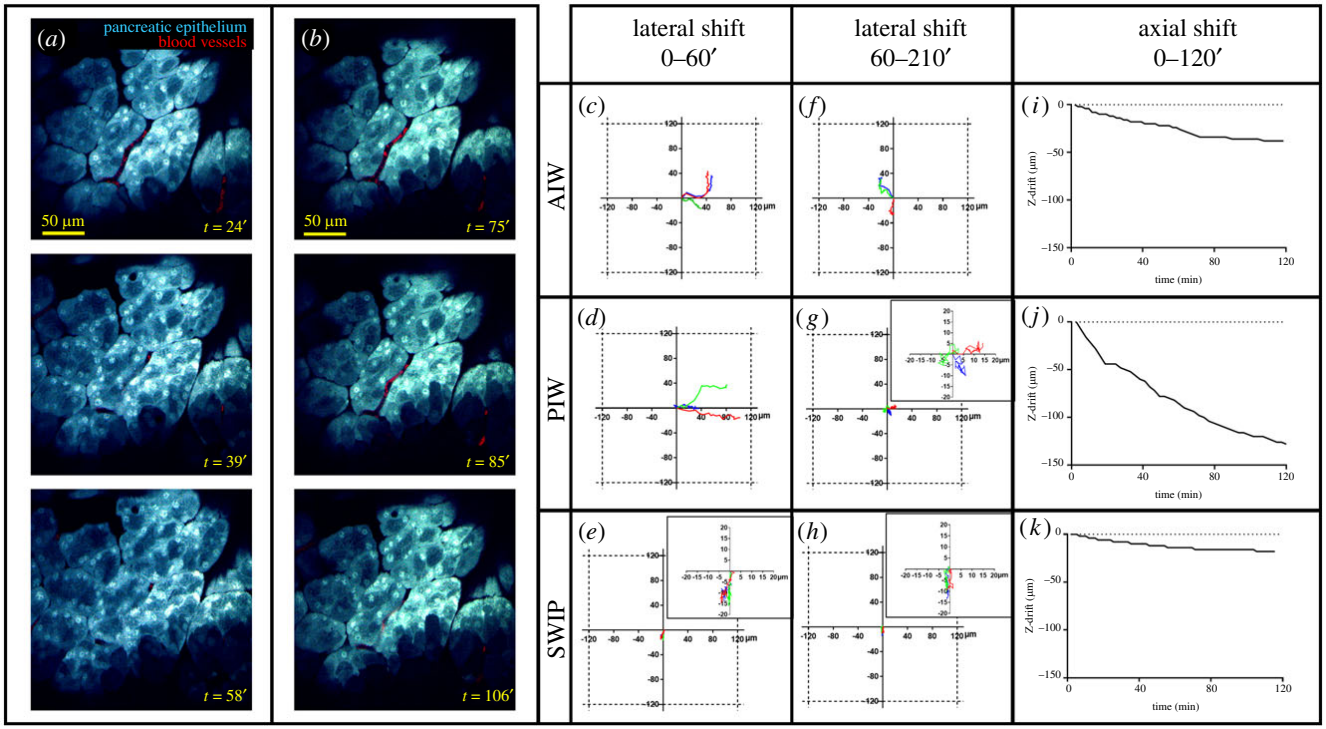
The SWIP greatly improves stability of imaging. (*a*) Stills from within the first 72′ of a time-lapse movie of the pancreas imaged through the SWIP. Some axial but very little lateral drift can be observed. See electronic supplementary material, movie 3. (*b*) Continued time-lapsed imaging after 72′ shows a high level of axial and lateral stability. See electronic supplementary material, movie 4. (*c*–*e*) Comparison of the lateral stability of each of the pancreas imaging windows during the first hour of imaging for the (*c*) abdominal imaging window (AIW), (*d*) pancreas imaging window (PIW), and (*e*) stabilized window for intravital imaging of the pancreas (SWIP). (*f*–*h*) Comparison of the lateral stability of each of the pancreas imaging during the subsequent 150 min for the (*f*) AIW, (*g*) PIW, and (*h*) SWIP. Insets are zoomed in views of the corresponding plots. (*i*–*k*) Comparison of the axial stability of each of the pancreas imaging windows for the first 120 min of imaging for the (*i*) AIW, (*j*) PIW and (*k*) SWIP.

In order to compare the performance of the SWIP to that of the AIW and the PIW, we implanted these windows into the abdomen of mice whose epithelia were transgenically labelled by cyan fluorescent protein [16] (see Methods). While we originally used these mice to label the breast epithelium, we found bright CFP expression within the pancreata as well. By tracking the motion of recognizable structures in the time-lapse movies, we were able to quantify the tissue stability over time (figure 2). We observed that each window required an initial period of ‘settling’ (approx. 1 h) during which a relatively larger degree of tissue motion was observed figure 2*a* and electronic supplementary material, movie 3. After this time, we observed an increased level of lateral and axial stability (figure 2*b*; electronic supplementary material, movie S4).

To directly compare the amount of drift experienced by each window, we quantified the amount of lateral and axial drift present in movies taken through the AIW, the PIW, and the SWIP by tracking an anatomical feature (such as a nucleus, or a blood vessel) over time in different mice. As shown in figure 2*c*–*e*, during the first hour, there was a period of ‘settling’ during which a high level of lateral drift was observed for the AIW and PIW (greater than 40 µm), while the lateral drift in the SWIP was slight (less than 16 µm). During the subsequent period, the PIW showed marked improvement in lateral stability and its drift was similar to the SWIP, while the AIW still performed poorly in immobilizing the tissue (figure 2*f*–*h*). Figure 2*i*–*k* shows the comparison of axial stability between these three kinds of imaging window. Though all windows exhibited the need for settling time, the SWIP showed the lowest level of drift overall and is suitable for long-term (up to the 12 h protocol limit) imaging (electronic supplementary material, movie S5).

To demonstrate the utility of the SWIP, we next used it for several applications. First, we visualized single cell dynamics in both the normal and diseased pancreas, as well as observing the morphologic changes that occur during the onset of chemically induced pancreatitis.

### 3.3. Application of the SWIP to imaging the healthy pancreas

**Figure 3.**
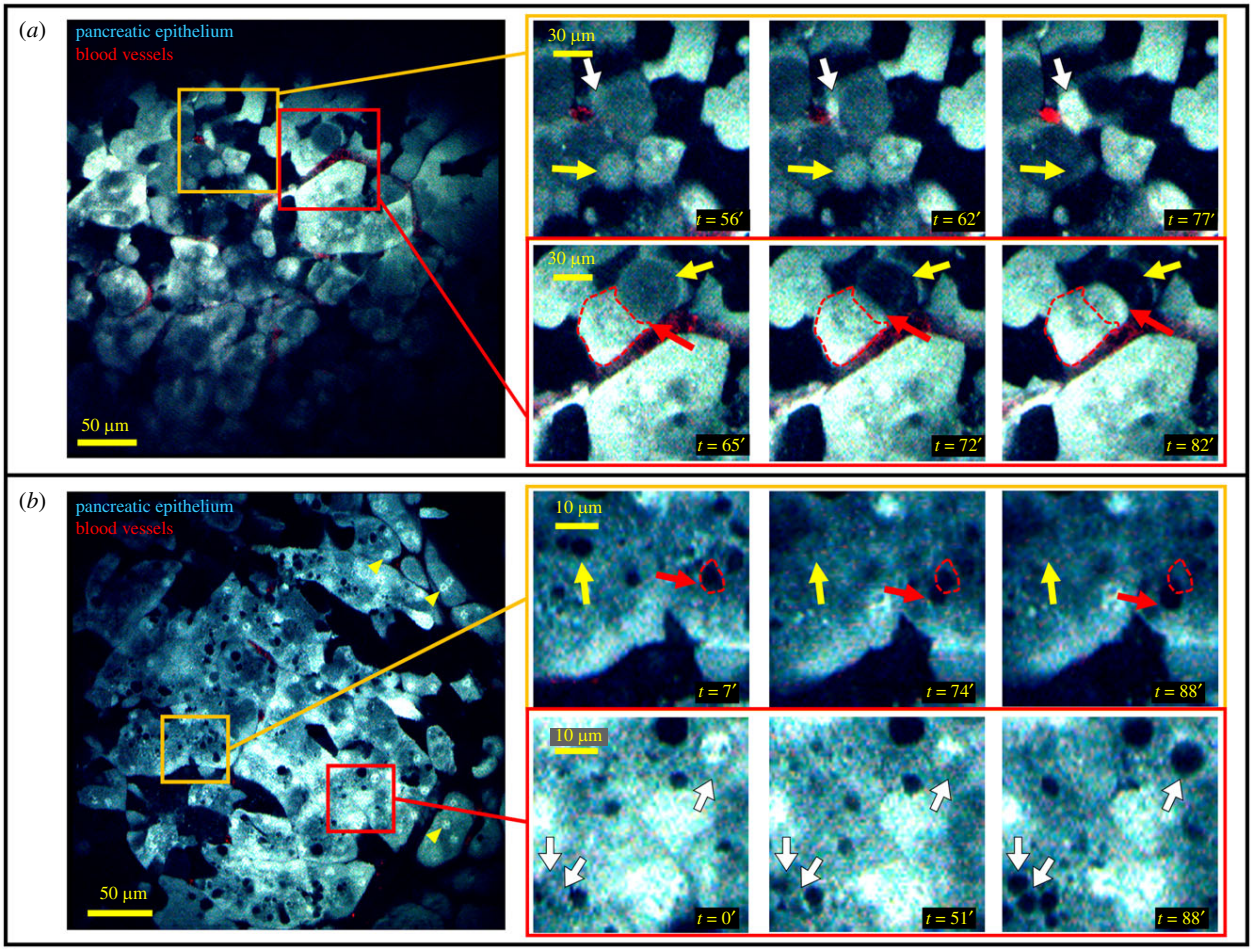
Cellular and subcellular dynamics in healthy pancreas. (*a*) In the healthy pancreas, motile acinar cells can be observed appearing (white arrows), disappearing (yellow arrows) and translocating to adjacent locations (red arrows, initial position of cell marked by red dashed outline). See electronic supplementary material, movie S7. (*b*) Subcellular structures such as nuclei are clearly visible (yellow arrow heads). Other subcellular structures that exclude the cyan fluorescent protein can be observed to appear (white arrows), disappear (yellow arrows), and translocate (red arrows, initial position marked by red dashed outline) over time. See electronic supplementary material, movie S8.

Given that the healthy pancreas has never before been visualized using single-cell resolution intravital imaging, we first implanted the SWIP into mice whose pancreatic epithelia were labelled by cyan fluorescent protein (see 'Material and methods'). Our expectation was that the cells of this mature and fully differentiated tissue would be static, as can be seen when imaging the ducts and lobules of the breast (electronic supplementary material, movie S6). Surprisingly, we found unexpected cell movement in the healthy pancreas with cells continually appearing, disappearing, and translocating during imaging (figure 3*a*; electronic supplementary material, movie S7). Appearance and disappearance of cells could not be accounted for by a shift in imaging plane as other structures such as blood vessels remained stationary in the images. In addition, many fields of view showed cells containing subcellular structures (figure 3*b*) such as nuclei and organelles (structures that exclude the CFP) that would also continually appear, disappear and translocate over time (figure 3*b*; electronic supplementary material, movie S8).

### 3.4. Application of the SWIP to cerulein induced acute pancreatitis

In order to study the onset of pancreatic disease, we used cerulein, an agent widely used in scientific research to induce acute pancreatitis [25–27]. Cerulein is a decapeptide with an amino acid sequence similar to cholecystokinin (CCK). As an analogue of CCK, it causes smooth muscle contraction of the gastrointestinal tract and pancreatic and gastric secretion [25]. When injected intraperitoneally, cerulein induces swelling and enlargement of the pancreas with concomitant increases of amylase concentration in the serum and active trypsin in the pancreatic tissue [26].

**Figure 4.**
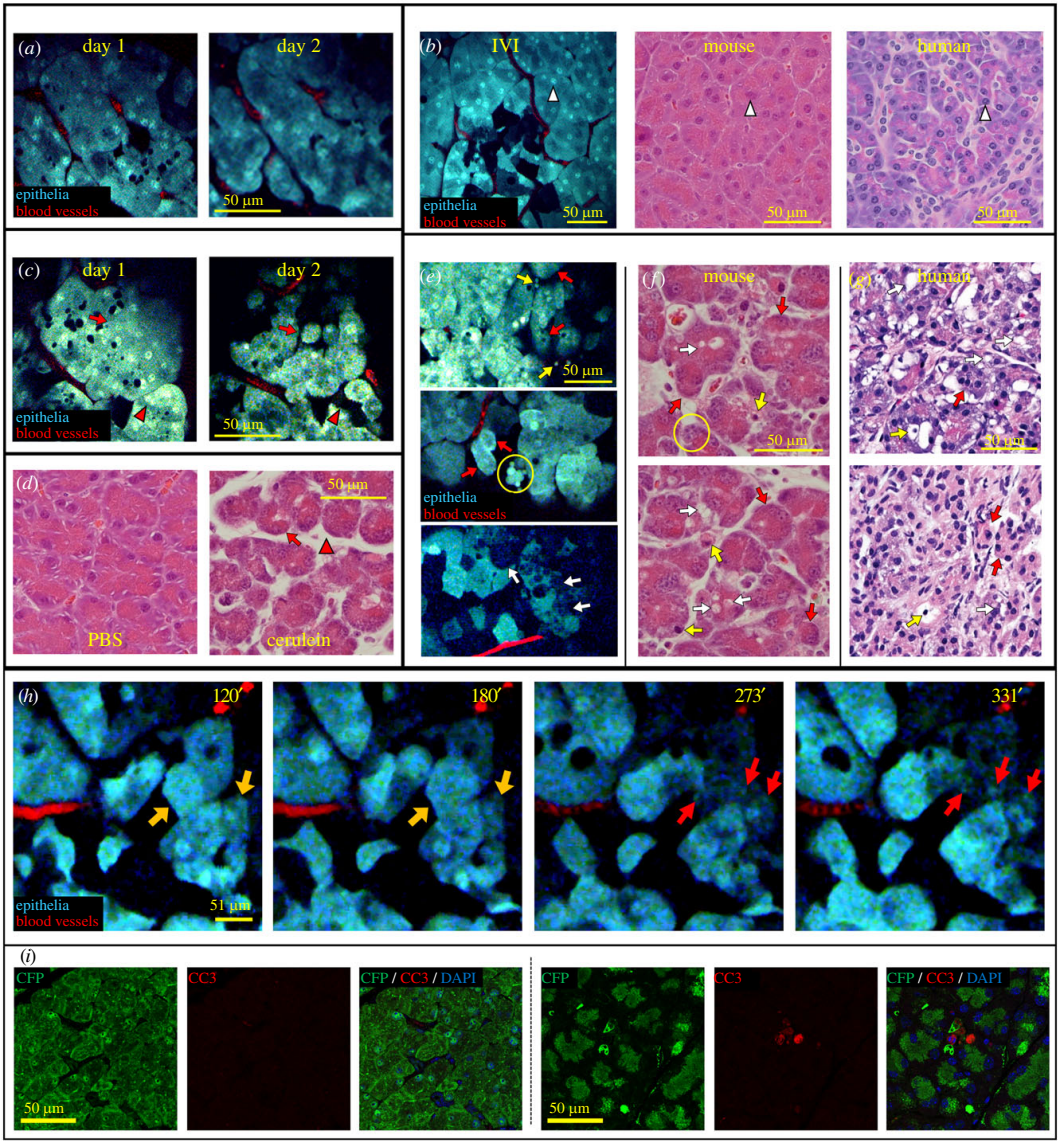
Cerulein treatment induces morphological changes. (*a*) Serial IVI of the pancreas over two consecutive days in mice treated with PBS. (*b*) Comparison of IVI and H&E images of healthy mouse (left and centre) and H&E image of the human pancreas (right) showing similar cellular morphology including pyramidal contours, round basally oriented nuclei (white arrowheads), and abundant granular cytoplasm. (*c*) Serial IVI of the pancreas over two consecutive days in mice treated with cerulein. Cell rounding (red arrows) is evident by 24 h after the first injection of cerulein. Cell shrinkage, presenting as intralobular space widening (red arrowheads) can also be observed. (*d*) H&E stained tissue sections from PBS and cerulein treated mice showing similar morphological changes of cell rounding (red arrow) and shrinkage (red arrowhead) within the cerulein treated pancreas. (*e*) IVI of cerulein-induced acute pancreatitis. Several features of pancreatitis appear visible including cell rounding (red arrows), apoptotic bodies (yellow arrows) and autolysosomes (yellow circle). Autophagic vacuolization in pancreatic cells could also be observed as time went by (white arrows). (*f*) Similar morphological changes can be observed in H&E sections of pancreas taken from identically treated mice, including cell rounding (red arrows), cell fragmentation or apoptotic bodies (yellow arrows), autolysosomes (yellow circle) and autophagic vacuoles (white arrows). (*g*) The changes observed in the mice reflect those observed in pancreatitis in human patients. These include cell rounding (red arrows), apoptotic bodies (yellow arrows), and autophagic vacuoles (white arrows). (*h*) Stills from a time-lapsed IVI movie showing the process of cell apoptosis due to acute pancreatitis induced by cerulein. Cytoplasmic CFP signal intensity can be seen to decreased gradually (orange arrows), then condense into separate apoptotic bodies (red arrows). See electronic supplementary material, movie S9. (*i*) Tissues taken from PBS- (left) and cerulein-treated (right) mice and stained for CFP (green), cleaved caspase-3 (CC3, red), a marker of cell apoptosis and DAPI (blue).

Given the permanent nature of the SWIP, we were able to use microcartography, our previously published field re-localization technique [7,24] to re-localize the same cells and image them multiple times to visualize the onset of acute pancreatitis. The pancreata of mice injected with PBS did not show significant morphological changes 24 h after treatment (figure 4*a*). The histological appearance of PBS treated mice was similar to untreated mice when viewed by IVI or H&E and morphologically similar to the healthy human pancreas (figure 4*b*).

However, in mice treated with cerulein, we observed a large number of histological changes commonly observed in samples of pancreatitis taken from rodents [28,29] and from patients [30], including cell rounding, autolysosomes, apoptotic bodies, autophagic vacuoles (electronic supplementary material, figure S2), as well as interstitial widening, indicative of interstitial edema (figure 4*c*). Similar histologic changes were seen by H&E staining (figure 4*d*). In addition to these changes, several other morphological changes indicative of cell death were able to be observed, including fluorescent fragments (clustered or in interlobular spaces) and intracellular vacuoles (figure 4*e*). Cell death due to photo-toxicity of the IVI can be ruled out, as this was observed without cerulein treatment, even in our 12 h imaging (electronic supplementary material, movie S5). Comparing these images with H&E stained slides of acute pancreatitis tissue in mice (figure 4*f*), the structures observed by IVI show the same morphology as histological changes previously identified as apoptotic bodies, autolysosomes and autophagic vacuoles [28–30]. Similar structures can be observed in tissue sections taken from patients suffering from acute pancreatitis (figure 4*g*). In addition to these snapshot images, IVI allows observation of the formation of these events in real time. As an example, figure 4*h* and electronic supplementary material, movie S7 show the formation of cellular fragments as an acinar cell apparently undergoes apoptosis. CFP signal intensity changes observed in the cytoplasm of these cells which points to cytoplasmic condensation and fragmentation into apoptotic bodies. This is consistent with what we observed when staining these tissues with cleaved caspase-3 (CC3), a marker of cell apoptosis. Pancreata taken from PBS-treated mice showed no appreciable level of CC3 staining (figure 4*i*, left), while rounded CC3 positive cells were evident in the cerulein-treated mice (figure 4*i*, right).

### 3.5. Application of SWIP to tumour-bearing mouse models

Given the high level of image stability provided by the SWIP, we were able to use our previously published technique for mosaicked acquisition of IVI movies, large-volume high-resolution intravital imaging (LVHR-IVI) [13]. In this method, time-lapsed, z-stack mosaics were built up by acquiring many individual, high-magnification tiles in a sequential manner, and stitching them together. Using this technique with tumour models is advantageous as it is able to provide better context to disorganized tissues while still capturing single-cell dynamics. As a proof of principle, we orthotopically implanted tumour cells from an immortalized tumour cell line (KPC) that expresses the fluorescent protein Dendra2 into MacBlue mice (where macrophages express a cyan fluorescent protein) [31] and, after 10–14 days, we implanted the SWIP.

**Figure 5.**
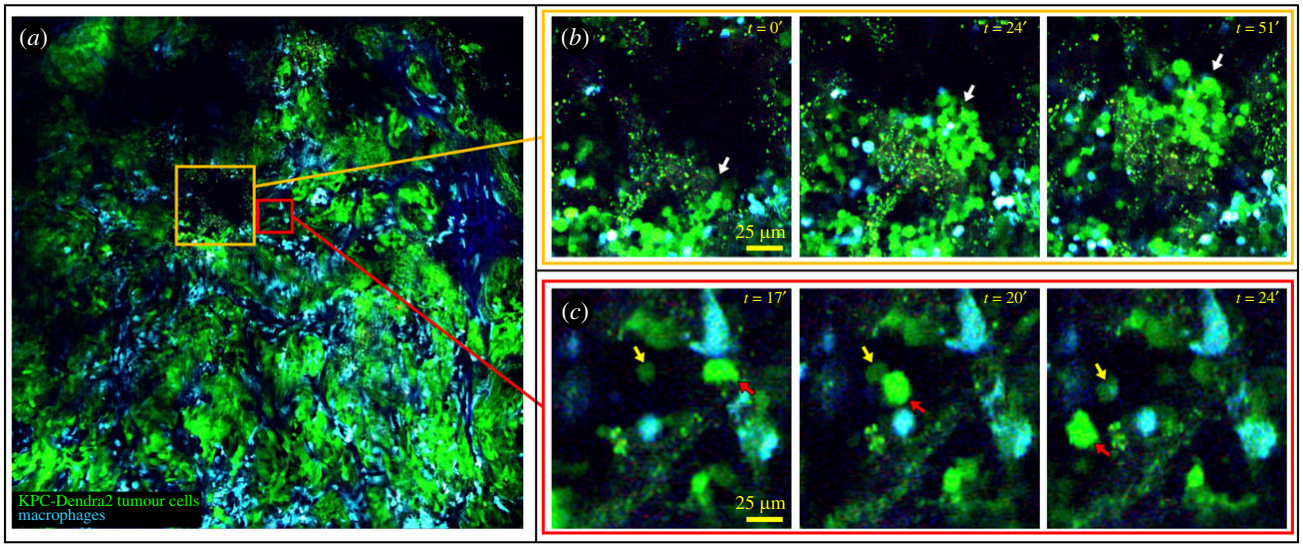
Application of the SWIP to orthotopically injected tumor mouse models. (*a*) Large-volume high-resolution intravital imaging (LVHR-IVI) captures time-lapse movies of large volumes of tumor tissue at single cell resolution and in real time. Left is a 4 × 4 mosaic covering 1.1 × 1.1 mm. Stills from simultaneously acquired sub-regions reveal single cell dynamics with subcellular resolution. See electronic supplementary material, movie S10. (*b*) An example of tumour cells undergoing collective migration. See electronic supplementary material, movie S11. (*c*) Examples of single cell migration (yellow and red arrows). Green = Dendra2 expressing tumour cells, Blue = CFP expressing macrophages. See electronic supplementary material, movie S12.

Using LVHR-IVI, we captured a 4 × 4 mosaic covering 1.1 × 1.1 mm of tumour tissue over a period of more than 1 h (figure 5; electronic supplementary material, movie S10). Our time-lapsed imaging showed several patterns of tumour cell movement including collective migration (figure 5*b*; electronic supplementary material, movie S11) and single cell migration (figure 5*c*; electronic supplementary material, movie S12).

Finally, we used the SWIP to image an autochthonous mouse model of PDAC (KPCY, containing mutations in Kras and p53 that are driven, along with YFP, by Pdx1-Cre expression in the pancreas) [18].

**Figure 6.**
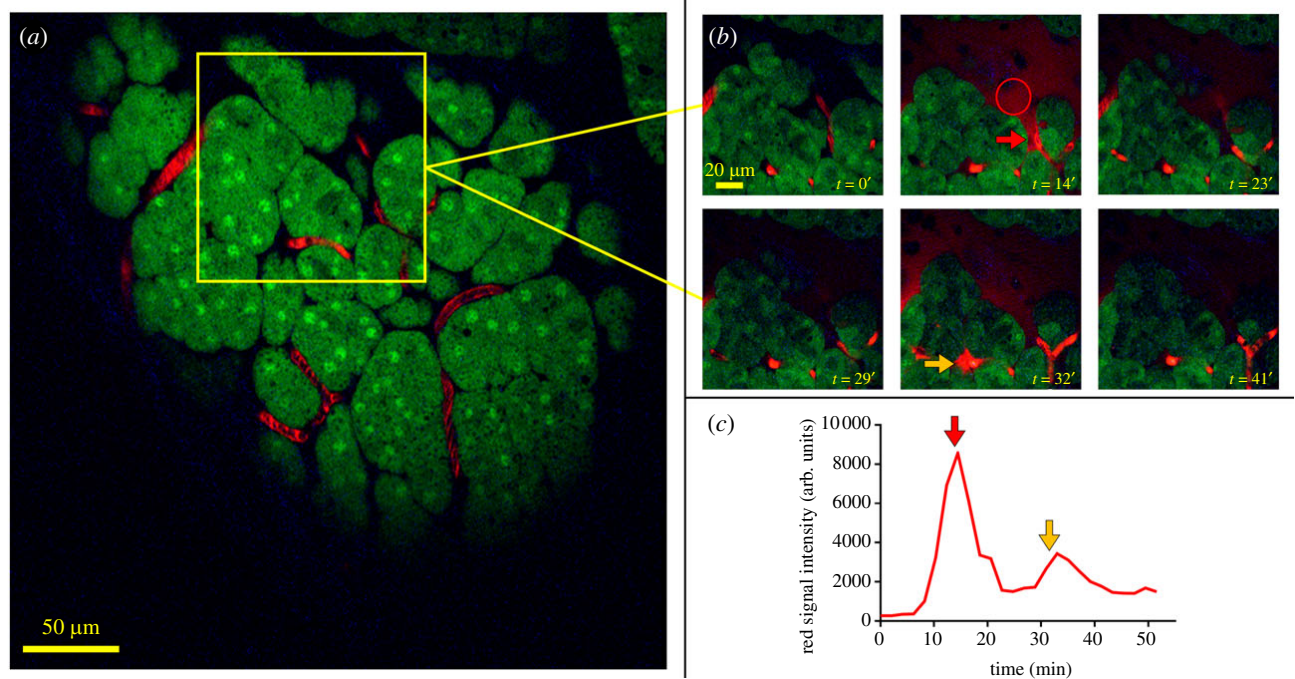
Application of the SWIP to spontaneously forming tumours in transgenic mouse models of cancer. (*a*) Still from a time-lapsed intravital imaging (IVI) movie of the pancreas in the transgenic mouse model of pancreatic ductal adenocarcinoma (PDAC). See electronic supplementary material, movie S13. (*b*) Stills from a time-lapsed IVI movie showing two transient vascular opening events (red and orange arrows). Red circle indicates area where signal intensity of the red channel (extravascular of 155-kD tetramethyl-rhodamine labeled dextran) was quantified. See electronic supplementary material, movie S12. (*c*) Plot of the signal intensity (region outlined by red circle in (*b*)) over time showing the dynamics of the transient vascular opening events. Red and orange arrows indicate the peaks of the first and second vascular opening events, respectively.

We IV injected KPCY animals with 155 kD TMR to label tumour vasculature and imaged their pancreata through the SWIP (figure 6*a*–*c*). As can be seen in figure 6*a*,*b* and electronic supplementary material, movies S13 and S14, we were able to observe the extravasation of serum contents through localized transient vascular openings followed by its rapid clearance. We observed this extravasation of dextran in the KPCY tumour bearing model (2 of 6 mice imaged) but not in non-tumor bearing mice (*n* = 5 mice: 2 PCY mice and 3 CY mice, electronic supplementary material, movie S15). The temporal dynamics of bursting (figure 6*c*) followed similar kinetics to those observed in mammary tumours [6].

## 4. Discussion

Intravital imaging has elucidated many physiological and pathological processes in various organs [32–36]. A prerequisite for successful intravital imaging is that the organ of interest to be exposed and stabilized for the duration of imaging. This is particularly challenging to accomplish for the pancreas, given its delicate composition, deep anatomical location and proximity to the diaphragm. To circumvent this challenge, a number of laboratories have investigated pancreatic processes *ex vivo* [37], with the pancreas being exteriorized from the body cavity [38], or using ectopic transplantation of tissues into sites more conveniently imaged, such as the eye [39], kidney [8] or subcutaneous tissue [40,41]. While recent advances have enabled intravital imaging of the pancreas, existing designs are still limited in either stability for long-term time-lapse imaging on the minutes time scale, or their ability to accommodate pancreata of a variety of sizes (e.g. healthy versus tumour bearing) [8,14,42].

Here, we present a new SWIP process, which improves long-term intravital imaging of both the healthy and tumour-bearing pancreata. In addition to comparing the performance of the SWIP to two other windows reported in the literature, we used the SWIP to image (1) single cell dynamics within the healthy pancreas, (2) transformation from healthy pancreas to acute pancreatitis induced by cerulein, and (3) physiology of PDAC in both orthotopically injected and autochthonous models. While the mechanical design of window frame used for the SWIP, and the use of a cross-stitch have been published before [15], neither have been applied to imaging of the pancreas and the surgical protocol required for implantation is completely different to address the unique anatomical location and physical characteristics of the pancreas. To our knowledge, this is the first study to visualize the live normal pancreas on the minutes to hours timescale or to observe the morphological changes incurred during the development of acute pancreatitis induced by cerulein, with intravital microscopy.

Indeed, within the healthy pancreas, we observed a surprising and previously unreported level of cellular and subcellular dynamics. Why these cells are motile, and why the pancreas, in particular, would show this motion is an intriguing question worthy of further investigation. Moreover, using serial imaging (where the same tissue can be visualized over multiple days (figure 4*a*,*c*) during the initial stages of pancreatitis in cerulein-treated mice, we could clearly observe the onset and progression of morphological changes that were previously reported using fixed tissues, including acinar cell autophagy (characterized by structures similar to cytoplasmic vacuoles and autolysosomes), cell apoptosis (characterized by nuclear and cytoplasmic condensation, and fragmentation of cytoplasma and nuclei), interstitial edema and cell shrinkage [26,28,29]. The power of the SWIP is demonstrated by directly observing these dynamic changes over time in the same tissue, and not correlated between different tissues. For example, individual cells undergoing apoptosis (which have been inferred to be the origin of apoptotic bodies, but could also have arisen through other bleb-inducing phenomena [43]) can be directly visualized with long-term time-lapsed microscopy (figure 4*h*; electronic supplementary material, movie S9).

The flexibility of the SWIP also allowed imaging of pancreata in tumour-bearing mice, both orthotopically injected, and those spontaneously forming within genetically manipulated mice. Consistent with the report by Beerling *et al*. [10], we did not observe collective migration of groups of cells that maintain cell–cell junctions in the spontaneous tumour model. Inconsistently, we also did not observe collective streams of single cells migrating together (streaming migration). This may be due to our use of tissues of a much earlier stage. However, in the orthotopically injected tumor model, both single cell and collective migration was present.

During our IVI of the murine pancreas in the KPCY mouse, we used high-molecular weight dextran to label the vasculature and incidentally observed extravasation of this agent into the interstitium of the pancreas. These bursting phenomena display the same temporal dynamics as was previously observed in primary breast tumours [6,44] and their metastases in the lungs [7] and lymph nodes [45]. In breast carcinoma, we have observed tumour cell intravasation occurs through entry points in the neoangiogenic vasculature called tumor microenvironment of metastasis (TMEM) doorways [6], consisting of a macrophage, a tumour cell and an endothelial cell all in direct contact [46–48]. We observed that during intravasation, the entry of tumour cells is accompanied by the transient opening of the vasculature and simultaneous escape of blood serum into the interstitium. Therefore, our observed bursting phenomena in these KPCY pancreata may be related to TMEM doorways. Further study will be needed to confirm that transient vascular openings in pancreatic tumours are associated with TMEM doorways and tumour cell intravasation.

Our work presents the first ever view of the dynamics of the pancreas at the minutes to hours timescale and at single-cell resolution, making all of the data presented novel biology and necessarily broad. This work is only a first foray into the unique, unanticipated biology of this organ. As with all intravital imaging techniques, the SWIP is limited in its ability to reveal information about cells and structures that are not explicitly labelled (e.g. the organelles we observed in the healthy pancreas), and to functionally manipulate proteins and cells while simultaneously visualizing them. Combination of windows such as the SWIP with fluorescent reporters and control of protein or cell states with pharmacologic and/or optogenetic tools should remove these limitations.

In summary, SWIP can be widely used in benign and malignant pancreatic diseases. With the improvement in the quality and stability of IVI, it can be a useful and promising tool to offer more information about the pathophysiology and cell biology of the pancreas.

## Data Availability

All data are available in the main text or the electronic supplementary material [49].
